# Flexible heuristic algorithm for automatic molecule fragmentation: application to the UNIFAC group contribution model

**DOI:** 10.1186/s13321-019-0382-3

**Published:** 2019-08-20

**Authors:** Simon Müller

**Affiliations:** Institute of Thermal Separation Processes, Hamburg University of Technology, Eißendorfer Straße 38, 21073 Hamburg, Germany

**Keywords:** Molecule fragmentation, Cheminformatics, RDKit, Property prediction, Group contribution method, UNIFAC, Incrementation

## Abstract

A priori calculation of thermophysical properties and predictive thermodynamic models can be very helpful for developing new industrial processes. Group contribution methods link the target property to contributions based on chemical groups or other molecular subunits of a given molecule. However, the fragmentation of the molecule into its subunits is usually done manually impeding the fast testing and development of new group contribution methods based on large databases of molecules. The aim of this work is to develop strategies to overcome the challenges that arise when attempting to fragment molecules automatically while keeping the definition of the groups as simple as possible. Furthermore, these strategies are implemented in two fragmentation algorithms. The first algorithm finds only one solution while the second algorithm finds all possible fragmentations. Both algorithms are tested to fragment a database of 20,000+ molecules for use with the group contribution model Universal Quasichemical Functional Group Activity Coefficients (UNIFAC). Comparison of the results with a reference database shows that both algorithms are capable of successfully fragmenting all the molecules automatically. Furthermore, when applying them on a larger database it is shown, that the newly developed algorithms are capable of fragmenting structures previously thought not possible to fragment.

**Electronic supplementary material:**

The online version of this article (10.1186/s13321-019-0382-3) contains supplementary material, which is available to authorized users.

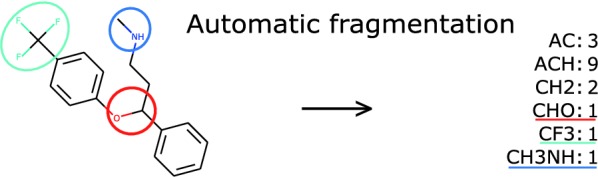

## Introduction

Cheminformatics is a growing field due to the increasing computational capabilities and improvements in the accuracy achieved by its predictions. The chemical space is vast and the number of molecules available to produce with new and, in some cases even automated synthetizing routes increases. However, before investing resources into synthetizing and characterizing molecules, a predictive approach for its properties would help narrow down the possible candidates. In addition, for the application of thermodynamic models or a priori calculation of thermophysical properties, predictive methods can be helpful and in some cases even necessary. These methods, which relate properties to the molecule structures are usually named QSPR methods (Quantitative Structure Property Relationship). One subgroup of these models is the group contribution method. The idea behind this method is to divide the value of a property of the complete molecule into its contributions based on the chemical groups or other molecular subunit. Group contribution models have been successfully applied to a wide variety of properties including density [1, 2], critical properties [3–5], enthalpy of vaporization [6], normal boiling points [7, 8], water–octanol partition coefficients [9–11], infinite dilution activity coefficients [12] and many more. Also, from Gibbs excess energy models [13–15] and equations of states [16–19] they provide an approach that allows widening their application range to molecules composed of the same chemical groups relatively easily.

However, in the development and application of these models a manual mapping of the groups has to be performed in most cases. This can hinder the fast development and testing of possible different group combinations, especially for larger number of molecules.

Jochelson [20], in 1968, already described a simple automatic routine for substructure counting. Most of research since [21–28] is focused more on describing algorithms for substructure search, ring perception and aromaticity perception. In a recent paper Ertl [29] proposed a new algorithm for automatic chemical group definition based on a large database. Fortunately, most of the current cheminformatic toolkits already include search and perception features, allowing to create new advanced fragmentation algorithms focusing on other problems.

One of the free tools offered online for structure analysis is Checkmol [28, 30]. It is an open-source program for finding a defined set of functional groups within a molecular structure. However, it checks its existence without counting the occurrence. Przemieniecki [31] developed an implementation of UNIFAC with automatic group fragmentation by means of a non-standardized way of specifying the fragmentation scheme. Some other free webpage services that allow a complete automatic fragmentation of molecules also exist, including the ones from the companies DDBST GmbH [32] and Xemistry GmbH [33]. In the first case, fragmentation is limited to the schemes supported by the webpage. In the second case, it is possible to provide own fragmentation rules allowing for fragmentation using different schemes. However, the terms of use only allow for a manual use of the website and without the ability to use the results in commercial applications. Furthermore, knowing how the algorithm works would allow to debug, find errors and improve it.

Tools that implement group contribution models like Octopus [34], thermo [35] or UManSysProp [36] would largely benefit from an improved flexible automated fragmentation algorithm based on standardized ways to define the fragmentation scheme that can handle complex molecules.

The goal of this work is to provide flexible algorithms that only need a simple fragmentation scheme based on the SMARTS language [37] which is easy to use for the rapid development and testing of group contribution methods on larger datasets.

## Challenges of automatic fragmentation

Several challenges like non-unique group assignment, incomplete group assignment and the composition of the fragmentation scheme itself can arise when developing an automatic fragmentation algorithm. These will be discussed in more detail in this section. The examples described are based on the fragmentation scheme from Table 1.

**Table 1:** Fragmentation scheme developed in this work for the published UNIFAC groups and the respective pattern described used for sorting

Group information			Descriptors							
Number	Name	SMILES	1	2	3	4	5	6	7	8
1	CH3	[CH3;X4]	False	False	1	True	0	False	0	0
2	CH2	[CH2;X4]	False	False	1	False	0	False	0	0
3	CH	[CH1;X4]	False	False	1	False	0	False	0	0
4	C	[CH0;X4], [CH0;X3]	False	False	1	False	0	False	0	0
5	CH2=CH	[CH2]=[CH]	False	False	2	True	0	False	0	1
6	CH=CH	[CH]=[CH]	False	False	2	False	0	False	0	1
7	CH2=C	[CH2]=[C], [CH2]=[c]	False	False	2	False	0	False	0	1
8	CH=C	[CH]=[CH0], [CH]=[cH0]	False	False	2	False	0	False	0	1
9	ACH	[cH]	False	False	1	False	0	True	0	0
10	AC	[cH0]	False	False	1	False	0	True	0	0
11	ACCH3	[c][CH3;X4]	False	False	2	False	0	True	0	0
12	ACCH2	[c][CH2;X4]	False	False	2	False	0	True	0	0
13	ACCH	[c][CH;X4]	False	False	2	False	0	True	0	0
14	OH	[OH]	False	False	1	True	1	False	0	0
15	CH3OH	[CH3][OH]	True	False	2	False	1	False	0	0
16	H2O	[OH2]	True	False	1	False	1	False	0	0
17	ACOH	[c][OH]	False	False	2	False	1	True	0	0
18	CH3CO	[CH3][CH0]=O	False	False	3	True	1	False	0	1
19	CH2CO	[CH2][CH0]=O	False	False	3	False	1	False	0	1
20	CH=O	[CH]=O	False	False	2	True	1	False	0	1
21	CH3COO	[CH3]C(=O)[OH0]	False	False	4	True	2	False	0	1
22	CH2COO	[CH2]C(=O)[OH0]	False	False	4	False	2	False	0	1
23	HCOO	[CH](=O)[OH0]	False	False	3	True	2	False	0	1
24	CH3O	[CH3][OH0]	False	False	2	True	1	False	0	0
25	CH2O	[CH2][OH0]	False	False	2	False	1	False	0	0
26	CHO	[CH][OH0]	False	False	2	False	1	False	0	0
27	THF	[CH2;R][OH0]	False	False	2	False	1	True	0	0
28	CH3NH2	[CH3][NH2]	True	False	2	False	1	False	0	0
29	CH2NH2	[CH2][NH2]	False	False	2	True	1	False	0	0
30	CHNH2	[CH][NH2]	False	False	2	False	1	False	0	0
31	CH3NH	[CH3][NH]	False	False	2	True	1	False	0	0
32	CH2NH	[CH2][NH]	False	False	2	False	1	False	0	0
33	CHNH	[CH][NH]	False	False	2	False	1	False	0	0
34	CH3N	[CH3][N], [CH3][n]	False	False	2	False	1	False	0	0
35	CH2N	[CH2][N]	False	False	2	False	1	False	0	0
36	ACNH2	[c][NH2]	False	False	2	False	1	True	0	0
37	C5H5N	n1[cH][cH][cH][cH][cH]1	True	False	6	False	1	True	0	0
38	C5H4N	n1[c][cH][cH][cH][cH]1, n1[cH][c][cH][cH][cH]1, n1[cH][cH][c][cH][cH]1	False	False	6	True	1	True	0	0
39	C5H3N	n1[c][c][cH][cH][cH]1, n1[c][cH][c][cH][cH]1, n1[c][cH][cH][c][cH]1, n1[c][cH][cH][cH][c]1, n1[cH][c][c][cH][cH]1, n1[cH][c][cH][c][cH]1	False	False	6	False	1	True	0	0
40	CH3CN	[CH3]C#N	True	False	3	False	1	False	1	0
41	CH2CN	[CH2]C#N	False	False	3	True	1	False	1	0
42	COOH	C(=O)[OH]	False	False	3	True	2	False	0	1
43	HCOOH	[CH](=O)[OH]	True	False	3	False	2	False	0	1
44	CH2Cl	[CH2]Cl	False	True	2	True	1	False	0	0
45	CHCl	[CH]Cl	False	True	2	False	1	False	0	0
46	CCl	[CH0]Cl	False	True	2	False	1	False	0	0
47	CH2Cl2	[CH2](Cl)Cl	True	False	3	False	2	False	0	0
48	CHCl2	[CH](Cl)Cl	False	True	3	True	2	False	0	0
49	CCl2	C(Cl)Cl	False	True	3	False	2	False	0	0
50	CHCl3	[CH](Cl)(Cl)Cl	True	False	4	False	3	False	0	0
51	CCl3	C(Cl)(Cl)(Cl)	False	True	4	True	3	False	0	0
52	CCl4	C(Cl)(Cl)(Cl)(Cl)	True	False	5	False	4	False	0	0
53	ACCl	[c]Cl	False	True	2	False	1	True	0	0
54	CH3NO2	[CH3][N+](=O)[O−]	False	False	4	True	3	False	0	1
55	CH2NO2	[CH2][N+](=O)[O−]	False	False	4	False	3	False	0	1
56	CHNO2	[CH][N+](=O)[O−]	False	False	4	False	3	False	0	1
57	ACNO2	[c][N+](=O)[O−]	False	False	4	False	3	True	0	1
58	CS2	C(=S)=S	True	False	3	False	2	False	0	2
59	CH3SH	[CH3][SH]	True	False	2	False	1	False	0	0
60	CH2SH	[CH2][SH]	False	False	2	True	1	False	0	0
61	Furfural	O=[CH]c1[cH][cH][cH]o1	True	False	7	False	2	True	0	1
62	DOH	[OH][CH2][CH2][OH]	True	False	4	False	2	False	0	0
63	I	[IH0]	False	True	1	True	1	False	0	0
64	Br	[BrH0]	False	True	1	True	1	False	0	0
65	CH#C	[CH]#C	False	False	2	True	0	False	1	0
66	C#C	C#C	False	False	2	False	0	False	1	0
67	DMSO	[CH3]S(=O)[CH3]	True	False	4	False	2	False	0	1
68	ACRY	[CH2]=[CH1][C]#N	True	False	4	False	1	False	1	1
69	Cl(C=C)	[$(Cl[C]=[C])]	False	True	3	True	1	False	0	0
70	C=C	[CH0]=[CH0]	False	False	2	False	0	False	0	1
71	ACF	[c]F	False	True	2	False	1	True	0	0
72	DMF	[CH](=O)N([CH3])[CH3]	True	False	5	False	2	False	0	1
73	HCON(CH2)2	[CH](=O)N([CH2])[CH2], [CH](=O)N([CH2])[CH3]	False	False	5	False	2	False	0	1
74	CF3	C(F)(F)F	False	True	4	True	3	False	0	0
75	CF2	C(F)F	False	True	3	False	2	False	0	0
76	CF	[C]F	False	True	2	False	1	False	0	0
77	COO	[CH0](=O)[OH0], [cH0](=O)[oH0]	False	False	3	False	2	False	0	1
78	SiH3	[SiH3]	False	False	1	True	1	False	0	0
79	SiH2	[SiH2]	False	False	1	False	1	False	0	0
80	SiH	[SiH]	False	False	1	False	1	False	0	0
81	Si	[Si]	False	False	1	False	1	False	0	0
82	SiH2O	[SiH2][OH0]	False	False	2	False	2	False	0	0
83	SiHO	[SiH][OH0]	False	False	2	False	2	False	0	0
84	SiO	[Si][OH0]	False	False	2	False	2	False	0	0
85	NMP	[CH3]N1[CH2][CH2][CH2]C(=O)1	True	False	7	False	2	False	0	1
86	CCl3F	C(Cl)(Cl)(Cl)F	True	False	5	False	4	False	0	0
87	CCl2F	C(Cl)(Cl)F	False	True	4	True	3	False	0	0
88	HCCl2F	[CH](Cl)(Cl)F	True	False	4	False	3	False	0	0
89	HCClF	[CH](Cl)F	False	True	3	True	2	False	0	0
90	CClF2	C(Cl)(F)F	False	True	4	True	3	False	0	0
91	HCClF2	[CH](Cl)(F)F	True	False	4	False	3	False	0	0
92	CClF3	C(Cl)(F)(F)F	True	False	5	False	4	False	0	0
93	CCl2F2	C(Cl)(Cl)(F)F	True	False	5	False	4	False	0	0
94	CONH2	C(=O)[NH2]	False	False	3	True	2	False	0	1
95	CONHCH3	C(=O)[NH][CH3]	False	False	4	True	2	False	0	1
96	CONHCH2	C(=O)[NH][CH2]	False	False	4	False	2	False	0	1
97	CON(CH3)2	C(=O)N([CH3])[CH3]	False	False	5	True	2	False	0	1
98	CONCH3CH2	C(=O)N([CH3])[CH2]	False	False	5	False	2	False	0	1
99	CON(CH2)2	C(=O)N([CH2])[CH2]	False	False	5	False	2	False	0	1
100	C2H5O2	[OH0;!$(OC=O);!R][CH2;!R][CH2;!R][OH]	False	False	4	True	2	False	0	0
101	C2H4O2	[OH0;!$(OC=O);!R][CH;!R][CH2;!R][OH], [OH0;!$(OC=O);!R][CH2;!R][CH;!R][OH]	False	False	4	False	2	False	0	0
102	CH3S	[CH3]S	False	False	2	True	1	False	0	0
103	CH2S	[CH2]S	False	False	2	False	1	False	0	0
104	CHS	[CH]S	False	False	2	False	1	False	0	0
105	MORPH	[CH2]1[CH2][NH][CH2][CH2]O1	True	False	6	False	2	False	0	0
106	C4H4S	[cH]1[cH][s;X2][cH][cH]1	True	False	5	False	1	True	0	0
107	C4H3S	[c]1[cH][s;X2][cH][cH]1, [cH]1[c][s;X2][cH][cH]1	False	False	5	True	1	True	0	0
108	C4H2S	[c]1[c][s;X2][cH][cH]1, [c]1[cH][s;X2][cH][c]1, [cH]1[c][s;X2][c][cH]1, [cH]1[c][s;X2][cH][c]1	False	False	5	False	1	True	0	0
109	NCO	N=C=O	False	False	3	True	2	False	0	2
118	(CH2)2SU	[CH2]S(=O)(=O)[CH2]	False	False	5	False	3	False	0	2
119	CH2CHSU	[CH2]S(=O)(=O)[CH]	False	False	5	False	3	False	0	2

In the name of the group, AC stands for aromatic carbon atom. The names of the groups are based on the original UNIFAC names as described on their webpage [44]. If several patterns were employed to find one group, these are shown separated by a comma. The underlined patterns were added to improve the matching of the algorithm in comparison to the results of the reference database. The values of the descriptors for each group, as described in “Simple fragmentation” section, are also shown in this table. For sorting, the boolean descriptor values can be replace by integer values (True: 1, False: 0). Descriptors: 1: Whether the pattern has zero bonds 2: Whether the pattern is simple 3: Number of atoms defining the group. 4: Whether the number of available bonds is one: first the patterns with one bond, then patterns with more bonds. 5: Number of atoms in the pattern that are neither hydrogen nor carbon. 6: Whether the pattern includes atoms in a ring. 7: Number of triple bonds. 8: Number of double bonds

### Non-unique group assignment

**Fig. 1:**
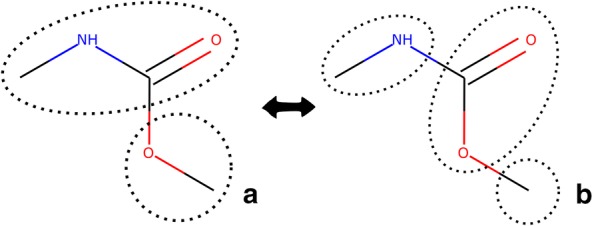
Example of a molecule with different functional groups where non-unique group assignment is possible. The groups identified are marked by the dotted line. Depending on where the algorithm starts to assign the groups, the result of the fragmentation is different. If the molecule is fragmented starting from left to right, the result might be the one shown in **a**, while if it is fragmented from right to left, the result might be as shown in **b**. SMILES: C[NH]C(=O)OC

For the assignment of the groups several solutions might be possible. The order in which the different groups are searched has an influence. For example, an ACOH group (hydroxyl bound to an aromatic carbon atom) can be recognized as such or fragmented into an aromatic carbon (AC) and a hydroxyl (OH) group. Furthermore, depending on the order in which the non-overlapping fragmentation is performed on the molecule structure, different results might be attained. For example, if a molecule is fragmented starting from left to right (Fig. 1a), the result obtained can be different from the one obtained if the molecule is fragmented from right to left (Fig. 1b).

In these cases, the algorithm must either deliver the correct fragmentation as a first solution or find all solutions and then specify how to choose the correct one.

### Incomplete group assignment

**Fig. 2:**
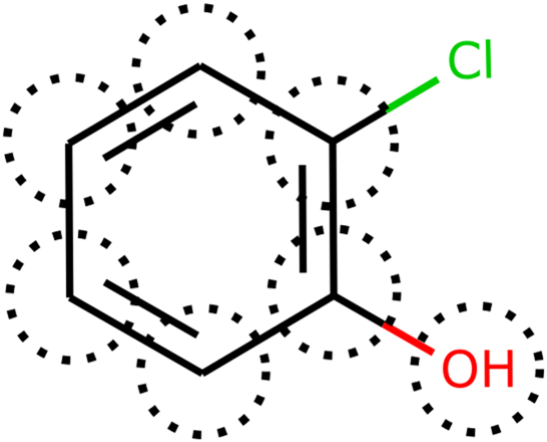
Example of a molecule with different functional groups where incomplete group assignment is possible. The groups identified are marked by the dotted line. The chlorine atom cannot be assigned to a group from the fragmentation scheme. SMILES: c1c(Cl)c([OH])ccc1

This case occurs when it is not possible to assign one or more atoms to a specific group. In some cases, the order of the groups searched can also lead to this situation. For example, in Fig. 2 if the AC groups (aromatic carbon) are searched first, the remaining chlorine atom cannot be assigned to any other functional group from the fragmentation scheme. In other cases, there will be molecules with atoms or functional groups that are just not defined in the fragmentation scheme. However, in most cases where the fragmentation is possible, this issue can be avoided if the algorithm specifies the order in which the functional groups are searched.

### The fragmentation scheme

Defining the fragmentation scheme is decidedly important for the accuracy of the algorithm. If the groups defined were targeting very specific functional groups or avoiding overlapping with other groups, this would minimize the non-unique or incomplete group assignments. A lot of time and testing can be invested in developing highly specific patterns for any given group contribution method such as those already done for UNIFAC by Salmina et al. [38]. However, if the algorithm includes a way to prioritize the groups from the fragmentation scheme, in most cases the groups do not have to be highly specific thus allowing to focus more time on developing different fragmentation schemes instead of refining one specific scheme.

## Strategies to overcome the challenges

To overcome the challenges described in the section “Challenges of automatic fragmentation”, three features were implemented in this work:

### Heuristic group prioritization

The patterns of the fragmentation scheme are sorted based on a set of heuristically determined descriptors. These descriptors can be, for example, the number of atoms describing the pattern, the number of bonds available or the number of double bonds.

### Parent–child group prioritization

The complete fragmentation scheme is analyzed to find patterns that are contained within others. E.g. CH2 is contained in CONHCH2. Whenever searching for a specific pattern, if the group has such a parent pattern, the parent pattern is searched first. After that, the child pattern is searched.

### Adjacent group search

To avoid incomplete group assignments, whenever a part of the structure is already fragmented, the subsequent matches have to be adjacent to the groups already found.

## The algorithms

There are two types of algorithms that are possible to fragment molecules. The first type of algorithm (simple fragmentation) searches for one possible solution and accepts the first one found. The second type of algorithm (complete fragmentation) tries to find all possible solutions to fragment the molecule. To achieve this, a full tree search on the complete structure over the entire fragmentation scheme has to be performed. Since more than one solution is inherently possible, a way should to be provided to prioritize the determined solutions and select one.

### Simple fragmentation

In the simple fragmentation algorithm, only one solution is searched. The patterns are sorted based on automatically calculated descriptors. In this work, the following set of 8 heuristically chosen descriptors were used to sort the patterns in descending order:

1. When the pattern has zero bonds: First, the patterns without bonds, then patterns with bonds are sorted.
2. When the pattern is simple: consisting of one atom with valence one or one atom with valence one connected to a carbon atom. First, the simple patterns, then the others are sorted.
3. Number of atoms defining the group: this number includes the atoms actually matched by the pattern as well as the ones defining the vicinity in case of recursive SMARTS.
4. When the number of available bonds is one: first, the patterns with one bond, then patterns with more bonds are sorted.
5. Number of atoms in the pattern that are neither hydrogen nor carbon.
6. When the pattern includes atoms in a ring: first the patterns that describe a partial ring (aliphatic or aromatic), then the other patterns are sorted.
7. Number of triple bonds.
8. Number of double bonds.

As a first step, the algorithm performs a quick search for the different groups in the fragmentation scheme applying the heuristic group prioritization and the parent–child group prioritization as described above. The search goes sequentially through the sorted fragmentation scheme, adding groups that are found and do not overlap with groups that were already found. In case it successfully finds a valid fragmentation, this is taken as the solution.

**Fig. 3:**
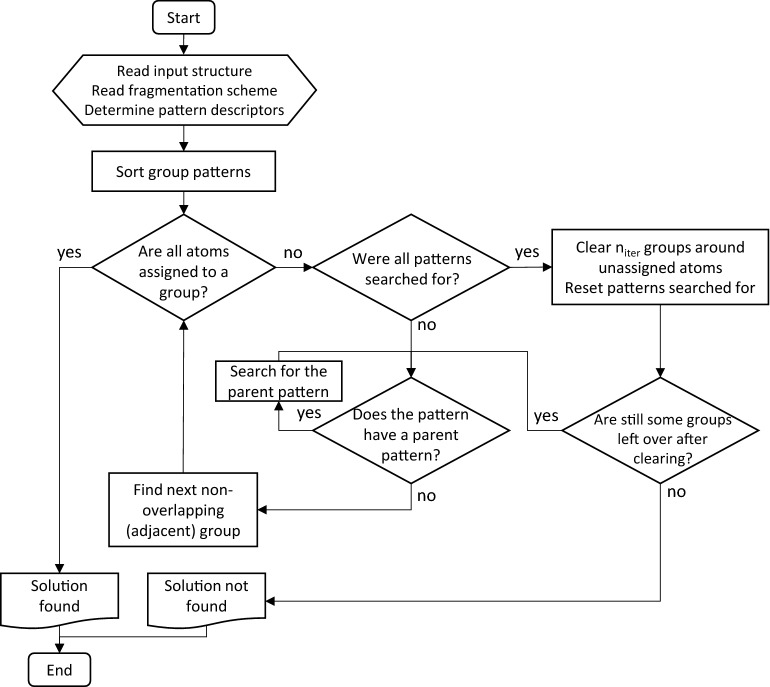
Schematic representation of the simple fragmentation algorithm

In case no solution is found after trying all fragmentation patterns, the area around the unassigned atoms is cleared of adjacent groups and the search is repeated applying all three features described above, i.e. searching only for non-overlapping groups that are contiguous to the groups already found. The clearing and searching might be repeated several times if no solution is found after the first iteration. In each subsequent iteration, a larger portion of the molecule connected to the unassigned atoms is cleared. If a valid fragmentation is found, this is taken as the solution. Figure 3 shows a flow-diagram-like schematic representation of the algorithm.

### Complete fragmentation

With the complete fragmentation algorithm, all possible solutions are searched. While the simple fragmentation algorithm might take milliseconds to find the fragmentation, the complete fragmentation algorithm might take minutes or even hours due to the vast space of possible combinations. Its search time increases exponentially with increasing molecule size. However, in contrast to the simple fragmentation, it allows to find all fragmentations and therefore its success in finding a solution is not dependent on the order of the searched patterns.

**Fig. 4:**
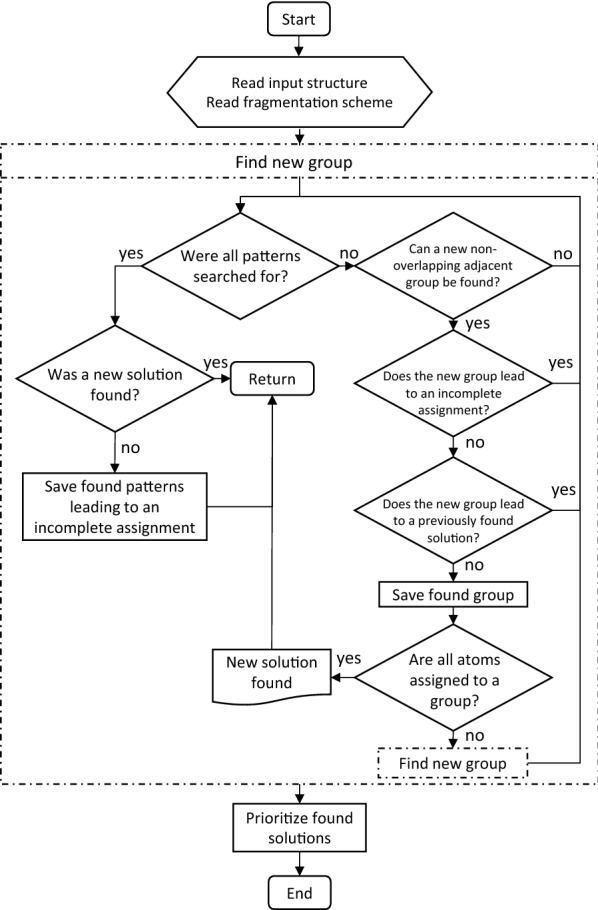
Schematic representation of the complete fragmentation algorithm

This algorithm was implemented as a recursive algorithm that performs a complete tree search of all possible combinations of fragmentation. To reduce the fragmentation space that needs to be searched, the algorithm keeps track of the solutions already found and of the group combinations that lead to an incomplete fragmentation. If several solutions were found in the end, the solutions were sorted by the number of different patterns and the first solution was taken as the determined fragmentation. This way, patterns with larger groups are prioritized over smaller patterns. Figure 4 shows a flow-diagram-like schematic representation of the algorithm.

## Computational details

In this work, the RDKit [39] python module was used to implement the algorithm. It supports the Simplified Molecular Input Line Entry System (SMILES) [40] and the SMiles ARbitrary Target Specification (SMARTS) [37] languages for specifying the molecular structures and the functional group patterns respectively. The SMARTS language is used as it provides a standardized, rich featured, easily learnable and wide spread approach to describe the molecular patterns.

To implement the parent–child group prioritization as described in “Parent–child group prioritization” section, it is necessary to test whether one pattern is contained within another. RDKit already works well when testing for most of the parent–child relationships. However, in some cases where the explicit amount of hydrogen atoms is important, the results are incorrect. For example, RDKit matches ‘[CH3][OH]’ as being contained in ‘[CH3][O;H0]’. Because of this, in this work, after a positive match the explicit amount of hydrogen atoms is tested to avoid false positives.

The research group of Computational Molecular Design at the University of Hamburg offers an online tool called SMARTSviewer [41, 42] that makes developing SMARTS patterns easier. This tool was used in the development process of the fragmentation scheme. The same group is also developing new algorithms to find the relationships between SMARTS patterns. In future, these developments might help improve the capabilities of cheminformatics modules such as RDKit to discern whether a pattern is contained within another.

The open source thermodynamics python module thermo [35] includes a large database of structures including single molecules and mixtures. After excluding salts and radicals, this comprises of a total set of 62,380 structures in the form of SMILES. For a subset of structures of this large database, fragmentations are available for use with the UNIFAC model. These structures were automatically fragmented using the service provided on the DDBST GmbH webpage [32]. This work first compares the results of the newly developed fragmentation algorithms with this reference database and then checks whether the new algorithms can fragment more structures than previously thought.

For some SMILES that include heavy versions of hydrogen, e.g. deuterium, these were replaced by normal hydrogen atoms. That makes 28,678 available SMILES with their corresponding UNIFAC fragmentation in the reference database.

For the sake of making the implementation of the algorithm easier in another group contribution model, the functions and the reference databases are made available as separate files in Additional files 1, 2, 3, 4 and on GitHub [43].

## Results and discussion

The fragmentation scheme for UNIFAC developed in this work can be found in Table 1. A version of the sorted fragmentation scheme according to the description in “Simple fragmentation” section can be found in Additional file 5.

The focus of this work is to develop a fragmentation algorithm that is as independent as possible from the chosen fragmentation scheme to allow for a faster development of new group contribution methods. For this reason, the SMARTS for each pattern were kept as simple as possible. The few patterns that were made more specific to match the results better from the literature database have been underlined. However, the overall majority of the SMARTS are as simple as they can be.

The fragmentation results are summarized in Table 2. Since the order of patterns searched can have an influence on the end result, both cases are differentiated in the table.

**Table 2:** Results of the fragmentation with both algorithms on the reference database

Algorithm	Sorted patterns?	N_SMILES_	N_fragmented_ (%)	N_likeRefDB_ (%)
Simple	Yes	28,678	28,677 (> 99.9%)	28,305 (98.7%)
Simple	No	28,678	18,969 (66.1%)	14,493 (50.5%)
Complete	Yes	24,336	24,335 (> 99.9%)	22,084 (90.7%)
Complete	No	24,336	24,335 (> 99.9%)	18,532 (76.1%)

For the complete algorithm, only the molecules with 20 or less heavy atoms were fragmented

**Fig. 5:**
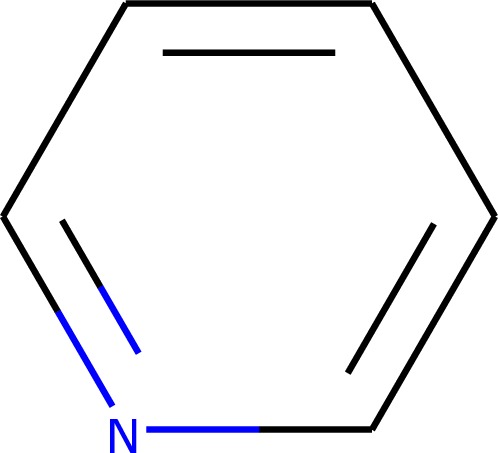
Only molecule that was not possible to fragment. SMILES: C1=CN=CC#C1

It can be observed that the simple fragmentation algorithm with the sorted patterns is able to fragment all but the molecule shown in Fig. 5. This is because there is no group in the fragmentation scheme matching the structure. The algorithm was able to fragment the molecules for every structure for which it should have been possible. This is a very encouraging result. Based on a set of general descriptors, by sorting the patterns automatically as much as 98.7% of the fragmented molecules match the fragmentation found by the algorithm from the reference database. Most of the remaining 1.3% of the fragmentations from the reference database can be explained by a different aromaticity perception. In the RDKit, a chemical bond is either described as being aromatic or being a single/double bond as opposed to the assignments done in the reference database where in some cases no distinction is made.

For the simple fragmentation algorithm, as expected, the sorting of the patterns plays a major role on the success of finding any solution at all and it is especially important to find the same solution as the reference database.

To evaluate the complete fragmentation algorithm only the molecules with 20 or less heavy atoms were included from the reference database. This was done because for very large molecules the algorithm takes hours to find all solutions.

Table 2 shows that since this algorithm searches for all possible fragmentations the amount of fragmented molecules is independent on whether the patterns are sorted or not. However, the results show that the sorting of the patterns has an influence on whether the chosen solution at the end is equal to the solution of the reference database.

This is because the order in which the different patterns is searched for defines the order of the found solutions from which the first one is selected.

The complete fragmentation algorithm could be refined further to sort the determined solutions at the end in a more elaborate way, for example, based on the descriptors of the patterns. However, this is out of the scope of this work.

Lastly, the algorithms were applied to the large database of structures included in thermo [35] to find out if the new algorithms are capable of fragmenting molecules that were not in the reference database. In this case, first the simple fragmentation algorithm was applied with the sorted patterns. If no solution was found with the simple fragmentation algorithm, the complete fragmentation algorithm was applied if the structure was smaller than 20 heavy atoms.

With this combined fragmentation algorithm, in total 33,560 structures were fragmented successfully. This number is 17% larger than the 28,677 fragmented structures in the reference database. This shows that the newly developed algorithms are capable of fragmenting more structures than the algorithm used in the reference database.

## Conclusions

Several challenges exist when attempting to fragment molecules into a set of predefined functional groups or molecular subunits. The strategies developed and implemented for the two algorithms in this work, show that it is possible to automate group fragmentation based on computed descriptors for the patterns in the fragmentation scheme. Both algorithms are capable of fragmenting every molecule of a reference database of structures into their respective UNIFAC groups. Furthermore, the algorithms are capable of fragmenting molecules that could not be fragmented by the algorithm of the reference database. The advancements of this work permit to accelerate the development of new group contribution models by allowing to test different fragmentations schemes on large databases of molecules much faster than with manual fragmentation, which is the existing standard for most group contribution models. It is a step forward in the direction of completely automated QSPR methods and maybe even completely automated group contribution development.

## Additional files

**Additional file 1.** Reference database of structures with fragmentations by the DDBST online fragmentation tool.

**Additional file 2.** Large database of structures without fragmentations by another method used to test the capability of the algorithms on more molecules.

**Additional file 3.** Code to reproduce results from the paper.

**Additional file 4.** Class encapsulating both algorithms for use in new applications.

**Additional file 5: Table S1.** Sorted fragmentation scheme developed in this work for the published UNIFAC groups and the respective pattern used for sorting.

## Data Availability

The datasets and the source code supporting the conclusions of this article are available in the GitHub repository, https://github.com/simonmb/fragmentation_algorithm_paper. Furthermore, these are also provided in Additional information to allow complete reproducibility of the work.
