# Supplementary material for: Flexible heuristic algorithm for automatic molecule fragmentation: application to the UNIFAC group contribution model
Source: J Cheminform. 2019 Aug 20;11:57. doi: 10.1186/s13321-019-0382-3 (PMC6701077; doi:10.1186/s13321-019-0382-3)
Supplement: Supplementary file 5 — Additional file 5: Table S1. Sorted fragmentation scheme developed in this work for the published UNIFAC groups and the respective pattern used for sorting. [file 13321_2019_382_MOESM5_ESM.docx]

**Additional Information for:**

**Flexible Heuristic Algorithm for Automatic Molecule Fragmentation: Application to the UNIFAC Group Contribution Model**

Simon Müller*

* corresponding author (simon.mueller@tuhh.de)

Hamburg University of Technology, Institute of Thermal Separation Processes, Eißendorfer Straße 38, D-21073 Hamburg, Germany

Table S1: Sorted fragmentation scheme developed in this work for the published UNIFAC groups and the respective pattern described used for sorting. In the name of the group, AC stands for aromatic carbon atom. The names of the groups are based on the original UNIFAC names as described on their webpage(44). If several patterns were employed to find one group, these are shown separated by a comma. The underlined patterns were added to improve the matching of the algorithm in comparison to the results of the reference database. The values of the descriptors for each group, as described in section 4.1, are also shown in this table. For sorting, the boolean descriptor values can be replace by integer values (True: 1, False: 0). Descriptors: 1: Whether the pattern has zero bonds 2: Whether the pattern is simple 3: Number of atoms defining the group. 4: Whether the number of available bonds is one: first the patterns with one bond, then patterns with more bonds. 5: Number of atoms in the pattern that are neither hydrogen nor carbon. 6: Whether the pattern includes atoms in a ring. 7: Number of triple bonds. 8: Number of double bonds.

| **Group Information** | | | **Descriptors** | | | | | | | |  |
| --- | --- | --- | --- | --- | --- | --- | --- | --- | --- | --- | --- |
| **Number** | **Name** | **SMILES** | **1** | **2** | **3** | **4** | **5** | **6** | **7** | **8** | |
| 61 | Furfural | O=[CH]c1[cH][cH][cH]o1 | True | False | 7 | False | 2 | True | 0 | 1 | |
| 85 | NMP | [CH3]N1[CH2][CH2][CH2]C(=O)1 | True | False | 7 | False | 2 | False | 0 | 1 | |
| 105 | MORPH | [CH2]1[CH2][NH][CH2][CH2]O1 | True | False | 6 | False | 2 | False | 0 | 0 | |
| 37 | C5H5N | n1[cH][cH][cH][cH][cH]1 | True | False | 6 | False | 1 | True | 0 | 0 | |
| 52 | CCl4 | C(Cl)(Cl)(Cl)(Cl) | True | False | 5 | False | 4 | False | 0 | 0 | |
| 86 | CCl3F | C(Cl)(Cl)(Cl)F | True | False | 5 | False | 4 | False | 0 | 0 | |
| 92 | CClF3 | C(Cl)(F)(F)F | True | False | 5 | False | 4 | False | 0 | 0 | |
| 93 | CCl2F2 | C(Cl)(Cl)(F)F | True | False | 5 | False | 4 | False | 0 | 0 | |
| 72 | DMF | [CH](=O)N([CH3])[CH3] | True | False | 5 | False | 2 | False | 0 | 1 | |
| 106 | C4H4S | [cH]1[cH][s;X2][cH][cH]1 | True | False | 5 | False | 1 | True | 0 | 0 | |
| 50 | CHCl3 | [CH](Cl)(Cl)Cl | True | False | 4 | False | 3 | False | 0 | 0 | |
| 88 | HCCl2F | [CH](Cl)(Cl)F | True | False | 4 | False | 3 | False | 0 | 0 | |
| 91 | HCClF2 | [CH](Cl)(F)F | True | False | 4 | False | 3 | False | 0 | 0 | |
| 67 | DMSO | [CH3]S(=O)[CH3] | True | False | 4 | False | 2 | False | 0 | 1 | |
| 62 | DOH | [OH][CH2][CH2][OH] | True | False | 4 | False | 2 | False | 0 | 0 | |
| 68 | ACRY | [CH2]=[CH1][C]#N | True | False | 4 | False | 1 | False | 1 | 1 | |
| 58 | CS2 | C(=S)=S | True | False | 3 | False | 2 | False | 0 | 2 | |
| 43 | HCOOH | [CH](=O)[OH] | True | False | 3 | False | 2 | False | 0 | 1 | |
| 47 | CH2Cl2 | [CH2](Cl)Cl | True | False | 3 | False | 2 | False | 0 | 0 | |
| 40 | CH3CN | [CH3]C#N | True | False | 3 | False | 1 | False | 1 | 0 | |
| 15 | CH3OH | [CH3][OH] | True | False | 2 | False | 1 | False | 0 | 0 | |
| 28 | CH3NH2 | [CH3][NH2] | True | False | 2 | False | 1 | False | 0 | 0 | |
| 59 | CH3SH | [CH3][SH] | True | False | 2 | False | 1 | False | 0 | 0 | |
| 16 | H2O | [OH2] | True | False | 1 | False | 1 | False | 0 | 0 | |
| 51 | CCl3 | C(Cl)(Cl)(Cl) | False | True | 4 | True | 3 | False | 0 | 0 | |
| 74 | CF3 | C(F)(F)F | False | True | 4 | True | 3 | False | 0 | 0 | |
| 87 | CCl2F | C(Cl)(Cl)F | False | True | 4 | True | 3 | False | 0 | 0 | |
| 90 | CClF2 | C(Cl)(F)F | False | True | 4 | True | 3 | False | 0 | 0 | |
| 48 | CHCl2 | [CH](Cl)Cl | False | True | 3 | True | 2 | False | 0 | 0 | |
| 89 | HCClF | [CH](Cl)F | False | True | 3 | True | 2 | False | 0 | 0 | |
| 69 | Cl(C=C) | [$(Cl[C]=[C])] | False | True | 3 | True | 1 | False | 0 | 0 | |
| 49 | CCl2 | C(Cl)Cl | False | True | 3 | False | 2 | False | 0 | 0 | |
| 75 | CF2 | C(F)F | False | True | 3 | False | 2 | False | 0 | 0 | |
| 44 | CH2Cl | [CH2]Cl | False | True | 2 | True | 1 | False | 0 | 0 | |
| 53 | ACCl | [c]Cl | False | True | 2 | False | 1 | True | 0 | 0 | |
| 71 | ACF | [c]F | False | True | 2 | False | 1 | True | 0 | 0 | |
| 45 | CHCl | [CH]Cl | False | True | 2 | False | 1 | False | 0 | 0 | |
| 46 | CCl | [CH0]Cl | False | True | 2 | False | 1 | False | 0 | 0 | |
| 76 | CF | [C]F | False | True | 2 | False | 1 | False | 0 | 0 | |
| 63 | I | [IH0] | False | True | 1 | True | 1 | False | 0 | 0 | |
| 64 | Br | [BrH0] | False | True | 1 | True | 1 | False | 0 | 0 | |
| 38 | C5H4N | n1[c][cH][cH][cH][cH]1,  n1[cH][c][cH][cH][cH]1,  n1[cH][cH][c][cH][cH]1 | False | False | 6 | True | 1 | True | 0 | 0 | |
| 39 | C5H3N | n1[c][c][cH][cH][cH]1,  n1[c][cH][c][cH][cH]1,  n1[c][cH][cH][c][cH]1,  n1[c][cH][cH][cH][c]1,  n1[cH][c][c][cH][cH]1,  n1[cH][c][cH][c][cH]1 | False | False | 6 | False | 1 | True | 0 | 0 | |
| 97 | CON(CH3)2 | C(=O)N([CH3])[CH3] | False | False | 5 | True | 2 | False | 0 | 1 | |
| 107 | C4H3S | [c]1[cH][s;X2][cH][cH]1,  [cH]1[c][s;X2][cH][cH]1 | False | False | 5 | True | 1 | True | 0 | 0 | |
| 118 | (CH2)2SU | [CH2]S(=O)(=O)[CH2] | False | False | 5 | False | 3 | False | 0 | 2 | |
| 119 | CH2CHSU | [CH2]S(=O)(=O)[CH] | False | False | 5 | False | 3 | False | 0 | 2 | |
| 73 | HCON(CH2)2 | [CH](=O)N([CH2])[CH2],  [CH](=O)N([CH2])[CH3] | False | False | 5 | False | 2 | False | 0 | 1 | |
| 98 | CONCH3CH2 | C(=O)N([CH3])[CH2] | False | False | 5 | False | 2 | False | 0 | 1 | |
| 99 | CON(CH2)2 | C(=O)N([CH2])[CH2] | False | False | 5 | False | 2 | False | 0 | 1 | |
| 108 | C4H2S | [c]1[c][s;X2][cH][cH]1,  [c]1[cH][s;X2][cH][c]1,  [cH]1[c][s;X2][c][cH]1,  [cH]1[c][s;X2][cH][c]1 | False | False | 5 | False | 1 | True | 0 | 0 | |
| 54 | CH3NO2 | [CH3][N+](=O)[O-] | False | False | 4 | True | 3 | False | 0 | 1 | |
| 21 | CH3COO | [CH3]C(=O)[OH0] | False | False | 4 | True | 2 | False | 0 | 1 | |
| 95 | CONHCH3 | C(=O)[NH][CH3] | False | False | 4 | True | 2 | False | 0 | 1 | |
| 100 | C2H5O2 | [OH0;!$(OC=O);!R][CH2;!R][CH2;!R][OH] | False | False | 4 | True | 2 | False | 0 | 0 | |
| 57 | ACNO2 | [c][N+](=O)[O-] | False | False | 4 | False | 3 | True | 0 | 1 | |
| 55 | CH2NO2 | [CH2][N+](=O)[O-] | False | False | 4 | False | 3 | False | 0 | 1 | |
| 56 | CHNO2 | [CH][N+](=O)[O-] | False | False | 4 | False | 3 | False | 0 | 1 | |
| 22 | CH2COO | [CH2]C(=O)[OH0] | False | False | 4 | False | 2 | False | 0 | 1 | |
| 96 | CONHCH2 | C(=O)[NH][CH2] | False | False | 4 | False | 2 | False | 0 | 1 | |
| 101 | C2H4O2 | [OH0;!$(OC=O);!R][CH;!R][CH2;!R][OH], [OH0;!$(OC=O);!R][CH2;!R][CH;!R][OH] | False | False | 4 | False | 2 | False | 0 | 0 | |
| 109 | NCO | N=C=O | False | False | 3 | True | 2 | False | 0 | 2 | |
| 23 | HCOO | [CH](=O)[OH0] | False | False | 3 | True | 2 | False | 0 | 1 | |
| 42 | COOH | C(=O)[OH] | False | False | 3 | True | 2 | False | 0 | 1 | |
| 94 | CONH2 | C(=O)[NH2] | False | False | 3 | True | 2 | False | 0 | 1 | |
| 41 | CH2CN | [CH2]C#N | False | False | 3 | True | 1 | False | 1 | 0 | |
| 18 | CH3CO | [CH3][CH0]=O | False | False | 3 | True | 1 | False | 0 | 1 | |
| 77 | COO | [CH0](=O)[OH0],  [cH0](=O)[oH0] | False | False | 3 | False | 2 | False | 0 | 1 | |
| 19 | CH2CO | [CH2][CH0]=O | False | False | 3 | False | 1 | False | 0 | 1 | |
| 20 | CH=O | [CH]=O | False | False | 2 | True | 1 | False | 0 | 1 | |
| 24 | CH3O | [CH3][OH0] | False | False | 2 | True | 1 | False | 0 | 0 | |
| 29 | CH2NH2 | [CH2][NH2] | False | False | 2 | True | 1 | False | 0 | 0 | |
| 31 | CH3NH | [CH3][NH] | False | False | 2 | True | 1 | False | 0 | 0 | |
| 60 | CH2SH | [CH2][SH] | False | False | 2 | True | 1 | False | 0 | 0 | |
| 102 | CH3S | [CH3]S | False | False | 2 | True | 1 | False | 0 | 0 | |
| 65 | CH#C | [CH]#C | False | False | 2 | True | 0 | False | 1 | 0 | |
| 5 | CH2=CH | [CH2]=[CH] | False | False | 2 | True | 0 | False | 0 | 1 | |
| 82 | SiH2O | [SiH2][OH0] | False | False | 2 | False | 2 | False | 0 | 0 | |
| 83 | SiHO | [SiH][OH0] | False | False | 2 | False | 2 | False | 0 | 0 | |
| 84 | SiO | [Si][OH0] | False | False | 2 | False | 2 | False | 0 | 0 | |
| 17 | ACOH | [c][OH] | False | False | 2 | False | 1 | True | 0 | 0 | |
| 27 | THF | [CH2;R][OH0] | False | False | 2 | False | 1 | True | 0 | 0 | |
| 36 | ACNH2 | [c][NH2] | False | False | 2 | False | 1 | True | 0 | 0 | |
| 25 | CH2O | [CH2][OH0] | False | False | 2 | False | 1 | False | 0 | 0 | |
| 26 | CHO | [CH][OH0] | False | False | 2 | False | 1 | False | 0 | 0 | |
| 30 | CHNH2 | [CH][NH2] | False | False | 2 | False | 1 | False | 0 | 0 | |
| 32 | CH2NH | [CH2][NH] | False | False | 2 | False | 1 | False | 0 | 0 | |
| 33 | CHNH | [CH][NH] | False | False | 2 | False | 1 | False | 0 | 0 | |
| 34 | CH3N | [CH3][N],  [CH3][n] | False | False | 2 | False | 1 | False | 0 | 0 | |
| 35 | CH2N | [CH2][N] | False | False | 2 | False | 1 | False | 0 | 0 | |
| 103 | CH2S | [CH2]S | False | False | 2 | False | 1 | False | 0 | 0 | |
| 104 | CHS | [CH]S | False | False | 2 | False | 1 | False | 0 | 0 | |
| 11 | ACCH3 | [c][CH3;X4] | False | False | 2 | False | 0 | True | 0 | 0 | |
| 12 | ACCH2 | [c][CH2;X4] | False | False | 2 | False | 0 | True | 0 | 0 | |
| 13 | ACCH | [c][CH;X4] | False | False | 2 | False | 0 | True | 0 | 0 | |
| 66 | C#C | C#C | False | False | 2 | False | 0 | False | 1 | 0 | |
| 6 | CH=CH | [CH]=[CH] | False | False | 2 | False | 0 | False | 0 | 1 | |
| 7 | CH2=C | [CH2]=[C],  [CH2]=[c] | False | False | 2 | False | 0 | False | 0 | 1 | |
| 8 | CH=C | [CH]=[CH0],  [CH]=[cH0] | False | False | 2 | False | 0 | False | 0 | 1 | |
| 70 | C=C | [CH0]=[CH0] | False | False | 2 | False | 0 | False | 0 | 1 | |
| 14 | OH | [OH] | False | False | 1 | True | 1 | False | 0 | 0 | |
| 78 | SiH3 | [SiH3] | False | False | 1 | True | 1 | False | 0 | 0 | |
| 1 | CH3 | [CH3;X4] | False | False | 1 | True | 0 | False | 0 | 0 | |
| 79 | SiH2 | [SiH2] | False | False | 1 | False | 1 | False | 0 | 0 | |
| 80 | SiH | [SiH] | False | False | 1 | False | 1 | False | 0 | 0 | |
| 81 | Si | [Si] | False | False | 1 | False | 1 | False | 0 | 0 | |
| 9 | ACH | [cH] | False | False | 1 | False | 0 | True | 0 | 0 | |
| 10 | AC | [cH0] | False | False | 1 | False | 0 | True | 0 | 0 | |
| 2 | CH2 | [CH2;X4] | False | False | 1 | False | 0 | False | 0 | 0 | |
| 3 | CH | [CH1;X4] | False | False | 1 | False | 0 | False | 0 | 0 | |
| 4 | C | [CH0;X4],  [CH0;X3] | False | False | 1 | False | 0 | False | 0 | 0 | |
